# Cardioprotective Effect of Selective Estrogen Receptor Modulator Raloxifene Are Mediated by Heme Oxygenase in Estrogen-Deficient Rat

**DOI:** 10.1155/2017/2176749

**Published:** 2017-07-09

**Authors:** Anikó Posa, Renáta Szabó, Krisztina Kupai, Anikó Magyariné Berkó, Médea Veszelka, Gergő Szűcs, Denise Börzsei, Mariann Gyöngyösi, Imre Pávó, Zoltán Deim, Zoltán Szilvássy, Béla Juhász, Csaba Varga

**Affiliations:** ^1^Department of Physiology, Anatomy and Neuroscience, Faculty of Science and Informatics, University of Szeged, Szeged, Hungary; ^2^Department of Cardiology, Medical University of Vienna, Vienna, Austria; ^3^Department of Pharmacology and Pharmacotherapy, Faculty of Medicine, University of Debrecen, Debrecen, Hungary

## Abstract

Estrogens and raloxifene (RAL) have beneficial effects on certain cardiovascular indices in postmenopausal women characterized by estrogen deficiency. Heme oxygenase (HO) activity is increased by 17*β*-estradiol (E_2_) and RAL in estrogen-deficient rat resulting in vasorelaxation mediated by carbon monoxide. We determined the expressions of HO in cardiac and aortic tissues after ovariectomy (OVX) and subsequent RAL or E_2_ treatment. We investigated the effects of pharmacological inhibition of HO enzyme on the arginine vasopressin- (AVP-) induced blood pressure in vivo, the epinephrine- and phentolamine-induced electrocardiogram ST segment changes in vivo, and the myeloperoxidase (MPO) enzyme activity. When compared with intact females, OVX decreased the HO-1 and HO-2 expression, aggravated the electrocardiogram signs of heart ischemia and the blood pressure response to AVP, and increased the cardiac MPO. E_2_ and RAL are largely protected against these negative impacts induced by OVX. The pharmacological inhibition of HO in E_2_- or RAL-treated OVX animals, however, restored the cardiovascular status close to that observed in nontreated OVX animals. The decreased expression of HO enzymes and the changes in blood pressure ischemia susceptibility and inflammatory state in OVX rat can be reverted by the administration of E_2_ or RAL partly through its antioxidant and anti-inflammatory roles.

## 1. Introduction

Although the clinical cardiovascular outcome study results in postmenopausal women are inconsistent and disappointing so far [1], estrogens demonstrated cardiovascular protective effects in various conditions and play an important role in the sex-related differences of hypertension in experimental models. Estrogen receptor-dependent and independent pathways result in favourable changes in plasma lipoproteins, haemostatic factors, glucose metabolism, and endothelium-derived factors as well as in the inhibition of smooth muscle cell migration and proliferation. Estrogen reduces both the myocardial infarct size and the occurrence of ischemia-reperfusion-induced damage and neutrophil infiltration in cardiac muscle [2]. In addition to their specific, receptor-mediated effects, estrogens have antioxidant properties also related to their aromatic/phenolic chemical structure: ovariectomy results in increased myeloperoxidase (MPO) enzyme activity [3, 4]. MPO acts as a master enzyme in the generation of reactive oxygen species (ROS) which promotes endothelial dysfunction by generating atherogenic-oxidized low-density lipoprotein OxLDL [5]. Elevated circulating MPO levels have been found to be associated with the presence of coronary artery disease (CAD) [6]. Estrogen replacement therapy has antioxidant properties and attenuates neutrophil infiltration and myeloperoxidase (MPO) activity in the heart [2].

Numerous studies prove that the cardioprotective effects of estrogens are mediated by the nitric oxide- (NO-) dependent pathway [7]. Similarly to NO, carbon monoxide (CO) also plays an important role in the estrogen-mediated cardioprotection. Equimolar amount of CO is generated during the catabolism of heme by the heme oxygenase (HO) enzymes. CO activates soluble guanylyl cyclase (sGC) by a mechanism similar to that for NO leading to smooth muscle relaxation. Three isoforms of HO have been characterized: HO-1, HO-2, and HO-3. HO-1 is widely expressed and can be induced by a host of stimuli that produces oxidative stress [8] and confers protection against vascular injury through its effects on constriction and proliferation against heart failure [9] and may play an important beneficial role in conditions such as hypertension and acute renal and lung injury [10, 11]. HO-2 occurs widely, including neuronal populations and vascular endothelial cells [12], and it is induced by glucocorticoids and probably estrogens [13, 14].

To overcome the adverse proliferative effects of estrogens on breast and endometrial tissues in the clinical practice, selective estrogen receptor modulators (SERMs) have been developed. Preclinical and clinical studies with RAL, a second-generation SERM, used for the prevention and treatment of postmenopausal osteoporosis, indicate its estrogen-like effects on the cardiovascular system. RAL improves the endothelial function in ovariectomized (OVX), aged, or hypertensive rats, ameliorates the hypertension-induced endothelial dysfunction by reducing the production of reactive oxygen species, and enhances endothelial nitric oxide- (NO-) dependent vasodilatation in vitro. Moreover, RAL causes direct vasodilatation [15]. It reduces the increased cardiovascular risk in patients with osteoporosis, although the outcomes of the RUTH trial showed that RAL did not affect the overall risk of coronary heart disease in elderly women. However, the incidence of coronary events was significantly lower in women < 60 years assigned to RAL compared with placebo. Measurements of cardiometabolic risk factors show that women assigned to RAL had greater increases in HDL cholesterol and greater reductions in LDL cholesterol, non-HDL lipoprotein levels, and the ratio of cholesterol to HDL, and fibrinogen levels. Moreover, a meta-analysis recently confirmed the beneficial effect of RAL administration on Lp(a) level [16].

The aim of this current study was to verify the extensive estrogen-agonist properties of RAL in cardiovascular system with analyzing of HO-1 and HO-2 isoforms. Therefore, we determined the effects of E_2_ and RAL treatments on the changes of blood pressure in vivo and ischemia susceptibility of the heart in adrenalin and phentolamine models. With pharmacological inhibition of HO, we evaluated its mediating role on these cardiac outcomes.

Epidemiological and clinical studies have shown a strong relationship between inflammatory markers and risk of future cardiovascular events. To examine how E_2_ replacement and RAL treatment change the inflammatory status of OVX rats, MPO activity was measured in myocardial tissue.

## 2. Materials and Methods

### 2.1. Examined Groups

**Figure 1:**
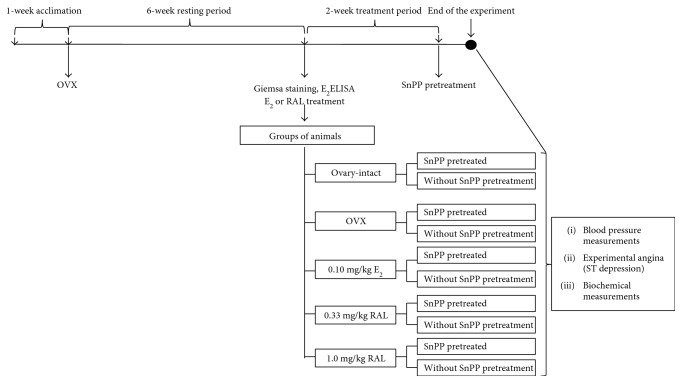
Experimental design of the study. OVX = ovariectomy, E_2_ = estrogen, RAL = raloxifene, SnPP = tin protoporphyrin IX.

4-month-old female Wistar rats (Laboratory Animals Producing Institute, Gödöllő, Hungary) were anesthetized and subjected to ovariectomy surgery (OVX). During OVX, the ovaries were clamped bilaterally and removed. After a 6-week resting period to verify the surgically induced menopause, the estrogen levels were checked by enzyme-linked immunosorbent assay according to the manufacturer's directions (Quantikine rat Estrogen ELISA kit, R&D Systems Inc.) [4]. Moreover, Giemsa staining was used to ensure that all animals were killed at the proestrus stage of the estrus phase. In separate groups of OVX animals, estrogen (estrofem, E_2_, 0.10 mg/kg/day, orally, once daily) or RAL (RAL 0.33: 0.33 mg/kg/day, RAL 1: 1.0 mg/kg/day, orally, once daily) replacement therapy was used for a 2-week period. HO activity was inhibited by tin protoporphyrin IX (SnPP, 30.0 *μ*g/kg, pH 7.4, s.c., 24 h and 1 h pretreatment). Each group consisted of at least 10 animals. All experimental procedures were performed in accordance with the standards of the European Community guidelines on the care and use of laboratory animals and had been approved by the Institutional Ethics Committee. The experimental design of the study is presented in Figure 1.

### 2.2. HO-1 and HO-2 Protein Expression

The aorta and cardiac left ventricle (LV) were homogenized in ice-cold Tris-mannitol and centrifuged for 20 min at 12,000*g* at 4°C. Protein content was measured by spectrophotometric assay. Aliquots of 25.0 *μ*g of total cellular protein were denatured and electrophoresed (100 V, 50 mA) on 10.0% polyacrylamide gel, transferred (100 V, 100 mA, 2 h) to nitrocellulose membrane, and then determined by staining the blot with 0.10% Ponceau red in 5.0% acetic acid. Two hours after blocking, the membranes were incubated with anti-HO-1 mouse monoclonal antibody (final dilution 1 : 1000) or anti-HO-2 monoclonal antibody (final dilution 1 : 1000) (StressGen Biotechnologies Corp., Victoria, Canada) for 2 h at room temperature, washed 3 times with PBS-Tween 20, and then exposed with horseradish peroxidase-conjugated bovine anti-mouse antibody (final dilution 1 : 2000; for 1 h at room temperature). Membranes were developed by using an enhanced chemiluminescence system and exposed to Hyperfilm. Films were analyzed by using ImageQuant Software after scanning with GelAnalyst 3.01 Software. The description of homogenization procedure, the content of solutions, as well as the producers of antibodies and equipment are detailed previously [17].

### 2.3. The Response of the Blood Pressure to AVP

Rats were anesthetized with 30.0% urethane and then pretreated with phentolamine (P, 10.0 mg/kg, i.p). After a stable baseline measurement, a single bolus injection of arginine vasopressin (AVP; 0.02, 0.06 or 0.18 *μ*g/kg) was infused intravenously to tail vein of rats. The first step of the procedure was to separate the right carotid artery, along with the vagus nerve, from the connective tissue. Then, the right carotid vessel was cannulated and the elevation of blood pressure was measured [18]. The cannula was connected to the pressor transducer, which converted the blood pressure into an electrical signal. To avoid a thrombotic process, the cannula was filled with 10.0% heparin. The changes in blood pressure were analysed by HAEMOSYS analysis system and expressed as a percentage of the maximal increase relative to basal value. We followed the methods of Posa et al. [17].

### 2.4. Experimental Angina Provoked by Epinephrine and Phentolamine

The standard limb lead II of the surface electrocardiogram (ECG) was recorded to measure the changes of ST segment by the HAEMOSYS system [19]. The changes in ST segment were used as the index of angina severity. During the specific experimental procedure, a single dose of epinephrine (10.0 *μ*g/kg) and 30 s later *α*-adrenoceptor antagonist P (15.0 mg/kg) were infused intravenously for 2 sec into the tail vein. After the administration of angina-provoking agents, the ST segment depression was calculated from the ECG waveform as a change in mV relative to the baseline level. We followed the methods of Posa et al. 2013 [17].

### 2.5. Cardiac MPO Activity

The cardiac tissues were homogenized in ice-cold PBS (pH 6.0), freeze-thawed three times, and then centrifuged twice at 15000*g* for 15 min at 4°C. The supernatant was discarded, and a 12 *μ*L aliquot was added to a mixture of 280 *μ*L of PBS (pH 6) and 0.167 mg mL^−1^ of O-dianisidine dihydrochloride. The reaction was started with 10 *μ*L of 0.03% hydrogen peroxide and assayed spectrophotometrically at 490 nm after 90 s of shaking. Cardiac MPO activity was expressed as mU/mg protein [20].

### 2.6. Chemicals

RAL (Eli Lilly and Company USA), AVP (Organon, The Netherlands), E_2_ (Novo Nordisc, Denmark), urethane (Reanal, Hungary), P (Ciba-Geigy, Switzerland), and SnPP (Frontier Scientific Europe, UK) were the chemicals used in this study. All compounds not specified above were derived from Sigma International.

### 2.7. Statistical Analysis

The results are expressed as means ± S.E.M. Western blots are shown as representative photographs of 3 independent experiments. Differences between groups were performed using ANOVA test, and *p* ≤ 0.05 was taken as significant.

## 3. Results

### 3.1. Actions of RAL or E_2_ Treatment on HO-1 and HO-2 Expression of LV and Aortic Tissues in Ovariectomized Rat

**Figure 2:**
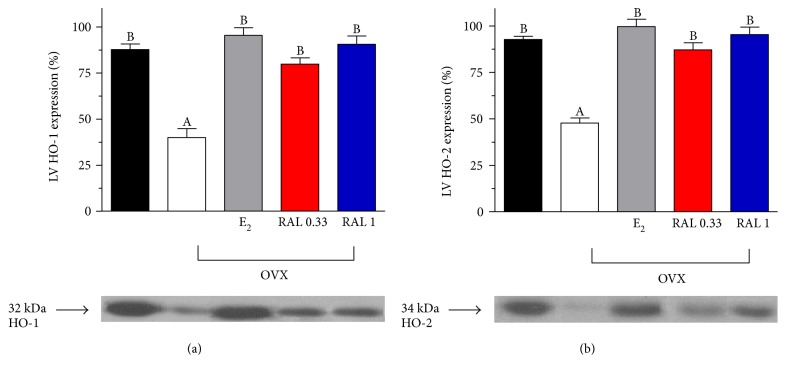
Heme oxygenase-1 and heme oxygenase-2 expression in the cardiac left ventricle. HO-1 (a) and HO-2 (b) expression (expressed as %) in the cardiac left ventricle (LV) of ovary-intact (black bar), ovariectomized (OVX (white bar)), and estrogen- (E_2_: (gray bar); 0.10 mg/kg/day, 2 weeks orally) or RAL-treated (RAL 0.33 (red bar): 0.33 mg/kg/day, RAL 1 (blue bar); 1.0 mg/kg/day, 2 weeks, orally) OVX rats. The diagrams demonstrate the densitometric assessment (means ± S.E.M. expressed as %; 100% is the maximal expression). Data are expressed as means ± S.E.M. of the results of a minimum of 10 rats per group. Statistical significance: (A) *p* < 0.001 as compared with the ovary-intact group. (B) *p* < 0.001 as compared with the OVX group without treatment.

Ovariectomy was found to lead to significantly decreased cardiac HO expression (HO-1: 39.86 ± 4.79%; HO-2: 48.0 ± 2.76%), and E_2_ (HO-1: 95.14 ± 4.11%; HO-2: 100.14 ± 4.02%) or RAL (RAL 0.33, HO-1: 79.5 ± 3.42%; HO-2: 87.55 ± 3.85%, RAL 1, HO-1: 90.29 ± 4.43%; HO-2: 95.86 ± 4.03%) supplementation in the OVX rats completely restored the HO expression to the level observed in the heart of the ovary-intact females. Data are shown in Figures 2(a) and 2(b).

**Figure 3:**
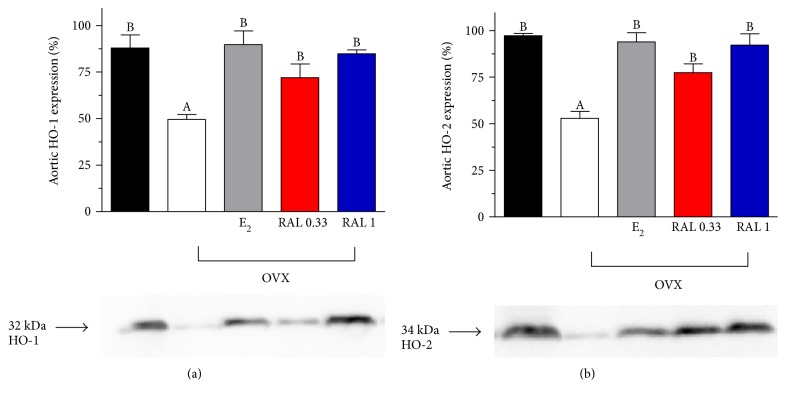
Heme oxygenase-1 and heme oxygenase-2 expression in the aorta. HO-1 (a) and HO-2 (b) expression (expressed as %) in the aortic tissues of ovary-intact (black bar), ovariectomized (OVX (white bar)), and estrogen-treated (E_2_: (gray bar); 0.10 mg/kg/day, 2 weeks orally), or RAL-treated (RAL 0.33 (red bar): 0.33 mg/kg/day RAL 1 (blue bar); 1.0 mg/kg/day, 2 weeks, orally) OVX rats. The diagrams demonstrate the densitometric assessment (means ± S.E.M. expressed as %; 100% is the maximal expression). Data are expressed as means ± S.E.M. of the results on a minimum of 10 rats per group. Statistical significance: (A) *p* < 0.001 as compared with the ovary-intact group. (B) *p* < 0.001 as compared with the OVX group without treatment.

Ovariectomy significantly decreased the aortic HO enzyme expression (HO-1: 49.86 ± 2.59%; HO-2: 53.0 ± 3.76%), and E_2_ (HO-1: 90.21 ± 7.41%; HO-2: 94.14 ± 5.02%) or RAL (RAL 0.33, HO-1: 72.34 ± 7.45%; HO-2: 77.55 ± 4.85%, RAL 1, HO-1: 85.31 ± 2.14%; HO-2: 92.46 ± 6.03%) supplementation in the OVX rats restored the HO expression. Data are shown in Figures 3(a) and 3(b).

### 3.2. The Effect of HO Inhibition on Blood Pressure as a Response to AVP

The arterial blood pressure was measured in the right carotid artery, and an increase was induced by i.v. administration of AVP (0.02–0.18 *μ*g/kg) in catecholamine-depleted (P, 10.0 mg/kg i.p.) female rats.

**Figure 4:**
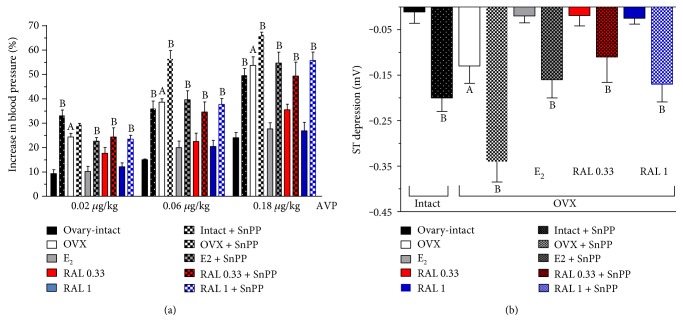
(a) The effect of HO inhibition on blood pressure as a response to AVP. The effects of HO inhibition by tin protoporphyrin IX (SnPP: 30.0 *μ*g/kg, pretreatment 24 h and 1 h prior to the measurement) on the increase in arterial blood pressure measured on administration of arginine vasopressin (0.02, 0.06, or 0.18 *μ*g/kg) in ovary-intact, ovariectomized (OVX), and estrogen- (E_2_: 0.10 mg/kg/day, 2 weeks orally, once daily) or RAL-treated (RAL 0.33: 0.33 mg/kg/day, RAL 1: 1.0 mg/kg/day, 2 weeks, orally, once daily) OVX rats. The intact + SnPP, OVX + SnPP, E2 + SnPP, RAL 0.33 + SnPP, and RAL 1 + SnPP columns show the actions of SnPP pretreatment (30 *μ*g/kg 24 h and 1 h prior to the measurement). Results are shown as means ± S.E.M. for 10 animals in each group. Statistical significance: (A) *p* < 0.05 compared with the ovary-intact group, and (B) *p* < 0.05 a significant difference between the groups with and without SnPP pretreatment. (b) The effect of inhibition of HO on ST depression. The effects of the heme oxygenase inhibitor tin protoporphyrin (SnPP) on the ST segment changes (measured in a lead II standard surface ECG; expressed in mV) following intravenous injection of epinephrine (10.0 *μ*g/kg) and 30 s later phentolamine (15.0 mg/kg) in ovary-intact, ovariectomized (OVX), and estrogen- (E_2_: 0.10 mg/kg/day, 2 weeks orally, once daily) or RAL-treated (RAL 0.33: 0.33 mg/kg/day, RAL 1: 1.0 mg/kg/day, 2 weeks, orally, once daily) OVX rats. The intact + SnPP, OVX + SnPP, E2 + SnPP, RAL 0.33 + SnPP, and RAL 1 + SnPP columns show the actions of SnPP pretreatment (30 *μ*g/kg 24 h and 1 h prior to the measurement). Results are shown as means ± S.E.M. for 10 animals in each group. Statistical significance: (A) *p* < 0.05 as compared with the ovary-intact group, and (B) *p* < 0.05 a significant difference between the groups with and without SnPP pretreatment.

AVP caused a dose-dependent increase in arterial blood pressure in both the ovary-intact and the OVX female rats. In the OVX animals, AVP induced a significantly higher elevation in blood pressure (24.30 ± 1.42 versus 53.60 ± 3.48%) than in the ovary-intact females (9.30 ± 1.62 – 24.0 ± 2.12%). Estrogen replacement (E_2_, 0.10 mg/kg, 2 weeks, orally, once daily) (10.20 ± 2.07 versus 27.60 ± 2.50%) abolished the increased blood pressure response, and RAL supplementation (RAL 0.33, 0.33 mg/kg, RAL 1; 1.0 mg/kg, 2 weeks, orally, once daily) (RAL 0.33: 17.6 ± 2.41–35.40 ± 2.30%, RAL 1: 12.10 ± 1.63–26.80 ± 3.45%) resulted in a decrease in the blood pressure enhancement provoked by AVP in the OVX rats. The inhibition of HO activity caused significant augmentation in all groups (ovary-intact: 33.10 ± 2.23% – 49.50 ± 2.77%; OVX group: 29.30 ± 0.56 – 66.10 ± 1.07%; E_2_-treated group: 22.60 ± 1.46 – 54.50 ± 4.50%; RAL-treated group: RAL 0.33: 24.0 ± 3.70% – 49.20 ± 5.78% RAL 1: 23.40 ± 1.60% – 55.60 ± 3.45%). Data are shown in Figure 4(a).

### 3.3. The Effect of Inhibition of HO on Cardiac Ischemia

ST segment changes were measured in a lead II standard surface ECG following i.v. injection of epinephrine (10.0 *μ*g/kg) and 30 s later phentolamine (15.0 mg/kg) in OVX female rats. The administration of phentolamine 30 s after epinephrine caused a significant ST segment depression only in the OVX rats (−0.13 ± 0.038 mV). In the ovary-intact females and in the E_2_- (0.10 mg/kg, 2 weeks, orally, once daily) or RAL-treated (1.0 mg/kg, 2 weeks, orally, once daily) OVX groups, an ST segment depression did not develop. Pretreatment with SnPP (30.0 *μ*g/kg, 24 h and 1 h prior to the measurement) caused a ST depression in the intact (−0.20 ± 0.03 mV) and E_2_ (−0.16 ± 0.04 mV) or RAL-treated (RAL 0.33: −0.11 ± 0.06 mV, RAL 1: −0.17 ± 0.04 mV) groups and augmented the ST depression in the OVX females (ST segment change: −0.34 ± 0.045 mV). Data are shown in Figure 4(b).

### 3.4. Cardiac Activity of MPO

**Figure 5:**
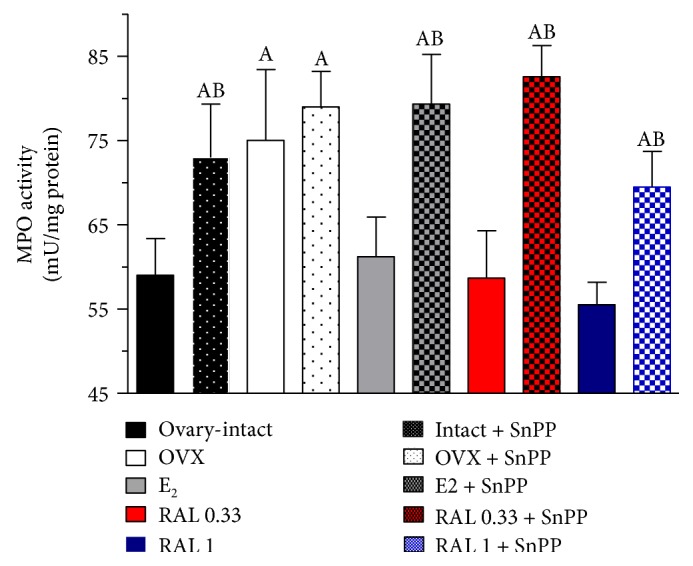
Myeloperoxidase activity in the cardiac left ventricle. Myeloperoxidase activity (MPO; expressed as mU/mg protein) in the cardiac left ventricle (LV) in ovary-intact, ovariectomized (OVX), and estrogen- (E_2_: 0.10 mg/kg/day, 2 weeks orally, once daily) or RAL-treated (RAL 0.33: 0.33 mg/kg/day, RAL 1: 1.0 mg/kg/day, 2 weeks, orally, once daily) OVX rats. The intact + SnPP, OVX + SnPP, E2 + SnPP, RAL 0.33 + SnPP, and RAL 1 + SnPP columns show the actions of SnPP pretreatment (30 *μ*g/kg 24 h and 1 h prior to the measurement). Data are expressed as means ± S.E.M. of the results on a minimum of 10 rats per group. Statistical significance: (A) *p* < 0.05 as compared with the ovary-intact group. (B) *p* < 0.05 as compared with the OVX group without treatment.

MPO activity was measured spectrophotometrically using *o*-dianisidine and hydrogen peroxide. In the OVX hearts, a significant increase in MPO activity was observed when compared with the ovary-intact females (75.0 ± 8.42–59.0 ± 4.37 mU/mg protein). Estrogen replacement therapy (E_2_, 0.10 mg/kg, 2 weeks, orally, once daily) and RAL treatment (RAL 0.33, 0.33 mg/kg, RAL 1; 1.0 mg/kg, 2 weeks, orally, once daily) caused a reduction in MPO activity of OVX groups (E_2_-treated group: 61.2 ± 4.69 mU/mg protein, RAL-treated group, RAL 0.33: 58.65 ± 5.63 mU/mg protein, RAL 1: 55.53 ± 2.64 mU/mg protein). Pretreatment with SnPP (30.0 *μ*g/kg, 24 h and 1 h prior to the measurement) significantly increased the MPO activity in the ovary-intact (59.0 ± 4.37–73.0 ± 6.34 mU/mg protein), E_2_-treated (61.2 ± 4.69–79.35 ± 5.86 mU/mg protein), and RAL-treated (RAL 0.33: 58.65 ± 5.63–82.56 ± 3.7 mU/mg protein, RAL 1: 55.53 ± 2.64–69.46 ± 4.24 mU/mg protein) groups. Data are shown in Figure 5.

## 4. Discussion

We have demonstrated cardiovascular protective features of E_2_ and RAL mediated by the HO system in OVX female rats. Estrogen depletion caused by ovariectomy was accompanied by a decreased expression of HO-1 and HO-2, elevated blood pressure, marked a ST segment depression, and increased MPO activity. These adverse effects could be markedly reversed by the exogenous administration of the E_2_ or RAL. These protections by E_2_ and RAL were partially offset by a pharmacological HO inhibitor, suggesting an important role of HO system in these findings.

Ovariectomy resulted in reduced HO-1 and HO-2 expression both in the LV of the heart and in the aorta. These data are in line with previous observations on the stimulatory effects of estradiol on the HO system [21]. E_2_ treatment elevated HO-1 protein levels and HO activity in trauma-hemorrhage male rats, resulting in the prevention of shock-induced organ damage [22]. Interestingly, in agreement with the present findings, HO-2, which is considered to be constitutively expressed, was also stimulated by E_2_ through an estrogen receptor-dependent mechanism in human endothelial cells [14]. Our results suggest that, together with glucocorticoids, E_2_ and RAL may belong to the few inducers of HO-2 [13]. Most inducers specifically act on HO-1. For example, hemin, a potent inducer of HO activity, increased HO-1, but not HO-2 expression in the mesenteric artery of young spontaneously hypertensive rats [23]. Similarly, lipopolysaccharides-induced HO-1, but not HO-2 mRNA expression in aortic tissues in rats [24]. We have previously reported that estrogen replacement and RAL treatment cause an increase in HO activity in OVX rat hearts and aorta [21]. In our recent study, we demonstrated that while estrogen deficiency reduces, estrogen supplementation restores HO expression in vivo.

We have found that, similarly to E_2_, RAL restores the HO expression in the heart and aorta of OVX rats. RAL induces HO-1 expression in mouse macrophages, resulting in inhibition of inducible NO synthase (iNOS) expression and the subsequent inflammatory reactions. However, these effects of RAL were not mediated by the estrogen receptor [25]. The inhibitory effect of RAL and estradiol on carrageenan-induced iNOS and acute inflammation in normal and OVX rats described earlier could probably also be mediated by HO induction [26]. Our results are the first demonstration that RAL also increases HO expression and activity in the cardiovascular system.

We found that OVX augmented the AVP-induced dose-dependent increase in blood pressure, as reported previously [18, 27]. E_2_ or RAL administration to OVX animals restored the blood pressure increase as compared with the control levels, irrespective of the AVP dose. These effects are at least partially mediated by the increased production of NO due to the constitutive nitric oxide synthase (cNOS) activity being elevated close to the pre-OVX level [18, 28]. In addition to the cNOS stimulation, the elevated HO activity induced by E_2_ or RAL also plays a role in the attenuated blood pressure response to AVP. The pretreatment with SnPP, a HO activity inhibitor, prevented the reduced blood pressure response by E_2_ or RAL. It is possible that the blood pressure responses in our model result from the interplay between the NOS and HO systems. Indeed, HO-1 overexpression restored endothelial NOS (eNOS) activity in endothelial cells under oxidative stress [29]. A low concentration of CO induced NO release, while a high concentration inhibited eNOS activity and NO generation [30]. Moreover, the potential nonspecific role of the selective HO inhibitor metalloporphyrins in vasoconstriction is also not fully elucidated. Certain other metalloporphyrins similar to SnPP may possess nonspecific vasoconstrictor effects in the rat small cerebral arteries, and SnPP could therefore possibly potentiate the blood pressure increase caused by HO inhibition [31]. In contrast, the metalloporphyrin, chromium mesoporphyrin, has been shown to increase the myogenic tone only of the small muscular branch of rat femoral arteries and not of large arterial vessels such as the aorta or the femoral artery [32]. Thus, the effects of the AVP-induced blood pressure increase in our experiments may largely represent reduced HO-1 and HO-2 activities, but we cannot exclude some additional direct effect by SnPP and the contribution of the interplay between the HO and NO systems. According to Ikeno et al., OVX caused significantly increased blood pressure response to AVP [27]. In our study, we found similar results. The AVP-induced blood pressure response in the presence of SnPP was also augmented in the sham-operated control animals. This in vivo finding supports the role of the basal, constitutive HO activity in the protection against vascular constriction found ex vivo; HO-1 knockout mice exhibited an impaired relaxation of the superior mesenteric arteries and an increased contractility to phenylephrine as compared with the vessels from wild-type animals [33, 34]. The HO inhibitor chromium mesoporphyrin increased the blood pressure in young spontaneously hypertensive rats [23]. Moreover, treatment with lipopolysaccharide induced the HO-1 and significantly reduced the blood pressure in rats, whereas pretreatment with the HO inhibitor zinc protoporphyrin-IX (ZnPP) prevented the fall in blood pressure [24]. Similarly, under stress conditions, while ZnPP decreased the aortic CO and cGMP levels, the acute vasoconstrictor effects of either *αα*Hb or NG-nitro-L-arginine methyl ester were restored in the rat after surgical intervention [35]. Previous studies have also demonstrated that either acute or chronic administration of various inducers of HO-1 to spontaneously hypertensive rats led to a normalization of the blood pressure [36]. In another model, the overexpression of HO-1 was associated with an increase in HO activity and a decrease in the blood pressure in spontaneously hypertensive rats [37]. In addition, Vera et al. demonstrated that induction of HO-1 decreases the blood pressure in angiotensin-II-dependent hypertension [38, 39].

In our study, estrogen deficiency increased the level of MPO. E_2_ substitution and RAL treatment, on the other hand, proved effective to attenuate the MPO activity in OVX rats. The connection between the MPO level and cardiac parameter or tissue HO expression suggests a preventive role of estrogen therapy in cardiovascular pathological processes. Similarly to our results, Chung et al. demonstrated that long-term treatment with RAL significantly decreased the cardiac activity of MPO in OVX rat [40]. While OVX increases the inflammation processes, the elevated levels of inflammatory markers can be decreased with hormone replacement therapy [41]. Oxidative stress plays a critical pathophysiological role during aging and after OVX. MPO is a major component of the oxidative system and displays potent proatherogenic properties. MPO can oxidize LDL cholesterol and reduces NO bioavailability, thereby impairing its vasodilatory and anti-inflammatory functions [42]. In our earlier study, we reported that higher levels of MPO have higher risk of cardiovascular events. The elevated level of the marker of leukocyte activation MPO correlated negatively with the tissue availability of cNOS and the indices of microvascular patency [43].

In conclusion, we have demonstrated that E_2_ supplementation and RAL treatment in OVX rats present beneficial effects on cardiovascular system, thereby increasing the HO-1 and HO-2 enzyme expression, decreasing the AVP-induced blood pressure, and attenuating the cardiovascular ischemia susceptibility. Estrogen administration has been shown to attenuate MPO activity in OVX rats.

Our study has several important limitations. Our experiments were performed in young OVX female rats. While this is a widely accepted estrogen deficient, “menopausal” rat model for the investigations of various conditions (hormone replacement therapies, cardiovascular health, osteoporosis, and so forth), our findings may not reflect adequately the situation in aging female rats and their relevance to postmenopausal women is even more limited. All cardiovascular changes and vasoconstrictions investigated in the present acute experiment reflect short-term alterations.
